# Spatial and temporal patterns of burn-related search interest in Brazil: a cross-sectional infodemiology study (2023–2024)

**DOI:** 10.1590/acb416726

**Published:** 2026-11-09

**Authors:** Wilson Falco, Guilherme Spuri Martins, Ricardo Baruque, Giovanna Cosin Ferreira da Silva, Bernardo Gabriele Collaco, Pedro Henrique Soubhia Sanches

**Affiliations:** 1 Faculdade de Medicina de Catanduva – Department of Plastic Surgery – Catanduva (SP) – Brazil.; 2 Mayo Clinic – Division of Plastic and Reconstructive Surgery – Jacksonville (FL) – United States.

**Keywords:** Burns, Infodemiology, Epidemiology

## Abstract

**Purpose:**

Burns are a leading cause of preventable morbidity globally, yet web search data’s value for burn surveillance is unclear. We investigated spatiotemporal associations between Google Trends interest and burn hospitalizations across Brazilian states.

**Methods:**

An ecological study covered all Brazilian states in 2023–2024, using the Department of Informatics of the Unified Health System/Hospital Information System (DATASUS/SIH) hospitalizations, Google Trends scores for “queimadura” (0–100 scale), and 2022 Brazilian Institute of Geography and Statistics’ census estimates. Spearman’s correlation, global Moran’s I, and SaTScan assessed correlation, autocorrelation, and temporal clustering.

**Results:**

A total of 64,380 burn hospitalizations occurred (32,075 in 2023; 32,305 in 2024), and incidence ranged from 0.47 to 53.77 per 100,000 inhabitants. Search interest correlated moderately with burn incidence in 2023 (ρ = 0.44; *p* = 0.02). Google Trends scores showed significant spatial autocorrelation in both years (2023: I = 0.36, *p* = 0.004; 2024: I = 0.25, *p* = 0.02), while hospitalization incidence did not. A temporal cluster occurred from June to October 2024 (relative risk = 1.08); related searches focused on treatment-seeking and first-aid.

**Conclusion:**

Search interest partially reflects the geographic and temporal burden of burns in Brazil, though inconsistently and independently of hospitalization patterns. Google Trends may complement burn surveillance, but findings warrant cautious interpretation pending further validation.

## Introduction

Burns are a major global public health challenge, causing an estimated 15.35 million cases in 2021^1^. In low- and middle-income countries, burns are influenced by social, economic, and educational factors, presenting higher incidence and mortality than in high-income nations^2^. In the Brazilian Unified Health System, burns are associated with prolonged hospitalizations, elevated morbidity, and expressive healthcare costs^3,4^.

Infodemiology has surged as a field in epidemiology that uses online search behavior to assess public health concerns^5^. Web search data platforms, such as Google Trends (GT), can evaluate the impact of health campaigns and provide early insights into emerging health threats^6-8^. As individuals seek health information online, they create digital footprints that may reflect population-level health concerns, information-seeking behaviors, and disease occurrence patterns^9^. Although an emerging epidemiological tool, the application of infodemiology to burn injury epidemiology remains limited despite its potential. Addressing this gap is important, as burn-related online search patterns may provide insights into population awareness of burn management and prevention. Brazil is a suitable setting for exploring burn infodemiology due to its large population^10^ and extensive number of internet users^11^.

This study examined the spatial and temporal links between GT search data for burns and burn hospitalizations across Brazilian states from 2023 to 2024. This study sought to explore web search data in burn epidemiology and surveillance, with implications for monitoring methods and public health practice.

## Methods

### Study design and ethical considerations

This ecological cross-sectional study was conducted in accordance with the Strengthening the Reporting of Observational Studies in Epidemiology (STROBE) guidelines^12^. The study utilized de-identified, publicly available data from open databases. Therefore, no institutional review board review was required per Brazilian ethical guidelines^13^. This protocol was pre-registered on the Open Science Framework prior to data analysis (10.17605/OSF.IO/WZC9S) ^14^.

### Data sources

Data were aggregated from three primary sources: Department of Informatics of the Unified Health System (DATASUS)^15^, GT^16^, and the Brazilian Institute of Geography and Statistics (IBGE)^17^.

Burn hospitalization data were obtained from the DATASUS/Hospital Information System (SIH) for the period from January 1, 2023, to December 31, 2024. Data were stratified by Brazilian state (n = 27, including the Federal District), sex, age group, and race/color. Data were extracted by the patient’s state of residence to ensure that incidence rates reflect geographic patterns of burn occurrence rather than healthcare facility location^15^.

Annual aggregate data were obtained for 2023 and 2024 separately, as well as for the combined 2023–2024 period. The GT score represents the normalized search interest on a 0–100 scale, and 100 indicates the peak search interest for the specified geographic region and time period^16^.

GT scores are dynamically normalized and may vary slightly between extractions of the same query. To minimize this variability, data were collected on a single date.

State population estimates were obtained from the IBGE’s 2022 census, the most recent national census available at the time of the analysis. Geographical data (shapefiles) for the Brazilian federation units were obtained from the IBGE for spatial analysis^17^.

The study period (2023–2024) was selected to avoid potential confounding from COVID-19 pandemic-related disruptions to healthcare-seeking behavior and hospitalization patterns. The year 2023 marks the official end of the COVID-19 public health emergency, as declared by the World Health Organization, and 2025 was not included to ensure complete data consolidation and reporting from DATASUS, as hospitalization data undergo a quality control and updating process before finalization^18^.

### Variables and data processing

Data were collected independently by two researchers in a blinded manner (W.F. and G.S.M.). A cross-check review was conducted in both raw format (immediately after download) and pre-processed format (prior to statistical analysis) to ensure data accuracy and consistency. Any discrepancies were resolved by consensus.

### Statistical analysis

#### Primary analysis

The association between GT scores and burn hospitalization incidence rates was evaluated using Spearman’s rank correlation coefficient, given the non-normal distribution of incidence data confirmed by the Shapiro-Wilk’s test. Two-tailed tests were conducted separately for 2023 and 2024 to assess the temporal consistency. All correlation analyses and data visualizations were performed using R (version 4.3; R Foundation for Statistical Computing, Vienna, Austria) within the RStudio integrated development environment (2023.06.0-421; Posit Software, PBC)^19^.

#### Secondary analysis

Spatial clustering was examined using the global Moran’s I statistic with a first-order Queen contiguity matrix and row-standardized spatial weights. Both univariate analyses (GT and hospitalization incidence rates independently) and bivariate spatial autocorrelation were performed to assess the degree to which high- or low-value states were spatially proximate to one another. The bivariate Moran’s was used to evaluate whether states with elevated burn incidence tend to be surrounded by neighbors with high search interest.

Statistical significance was assessed using 9,999 Monte Carlo permutations, with results considered significant at a pseudo *p* < 0.05. All spatial analyses were conducted using GeoDa (version 1.22; GeoDa Center for Geospatial Analysis and Computation, Chicago, IL, United States of America)^20^.

#### Exploratory analysis

Temporal clustering of burn hospitalizations across the 2023–2024 study period was assessed using purely temporal scan statistics implemented in SaTScan (version 10.1; Information Management Services, Rockville, MD, United States of America)^21^ under a discrete Poisson probability model. Both high- and low-risk clusters were evaluated, with a minimum cluster duration of one month and a maximum temporal window of 50% of the total study period. The null hypothesis of spatiotemporal randomness was tested using 9,999 Monte Carlo replications, and clusters were considered statistically significant at *p* < 0.05.

Choropleth maps for GT scores and hospitalization incidence were generated using QGIS Geographic Information System (QGIS.org Association, Switzerland; version 3.44)^22^. Color-blind-friendly alternatives are provided in the Suppl. Mat.^23^, along with an additional description of the methods.

## Results

Complete data were available for all 27 Brazilian states across 2023–2024, with no missing values for burn hospitalizations, GT scores, or population estimates. GT scores for “queimadura” ranged from 66 to 100 in both years, with mean scores of 82.00 (standard deviation [SD] = 9.00) in 2023 and 84.44 (SD = 10.00) in 2024 (Fig. 1).

**Figure 1:**
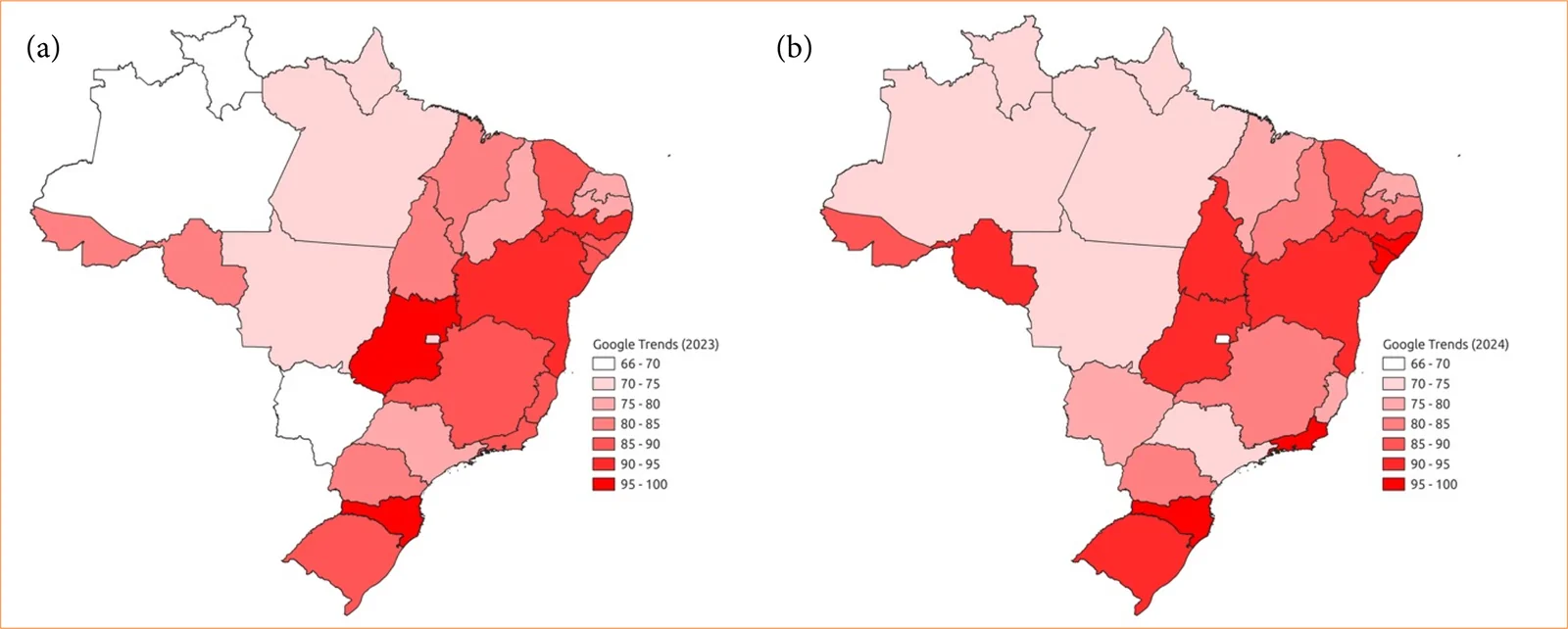
Choropleth maps showing the distribution of Google Trends scores (0–100) across Brazilian states in (a) 2023 and (b) 2024. Darker red shades indicate higher relative search interest. Map boundaries delineate study areas and do not necessarily reflect accepted national borders.

Source: Elaborated by the authors using data from Google Trends^16^ and IBGE^17^.

During the study period (2023–2024), 64,380 burn hospitalizations were recorded nationally (32,075 in 2023 and 32,305 in 2024). Stratified hospitalizations are described in the Suppl. Mat.^23^. Burn hospitalization incidence rates ranged from 0.47 to 53.77 per 100,000 inhabitants (mean = 15.44, SD = 10.03) in 2023 and from 1.10 to 50.69 per 100,000 (mean = 15.89, SD = 9.72) in 2024. The three states with the highest burn incidence in 2023 were Goiás (53.77 per 100,000), Pernambuco (32.12), and Paraná (25.69). The highest burn incidence in 2024 were Goiás (50.69), Pernambuco (31.23), and Paraná (26.31) (Fig. 2). Color-blind-accessible figures for the distribution of scores and hospitalizations are provided in the Suppl. Mat.^23^.

**Figure 2:**
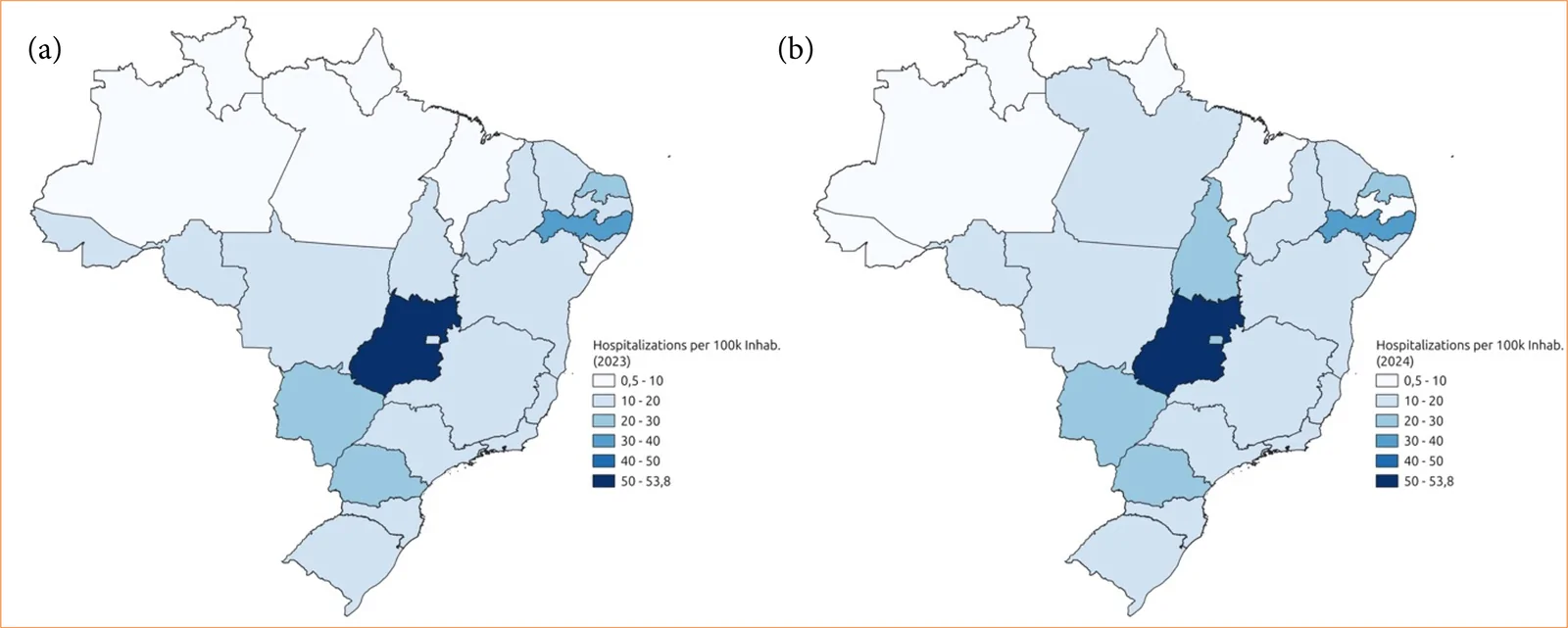
Choropleth maps showing burn hospitalization incidence rates (per 100,000 inhabitants) across Brazilian states in (a) 2023 and (b) 2024. Darker blue shades indicate higher incidence. Map boundaries delineate study areas and do not necessarily reflect accepted national borders.

Source: Elaborated by the authors using data from DATASUS/SIH^15^ and IBGE^17^.

Correlation analysis revealed a moderate positive association between GT scores and burn hospitalization incidence in 2023 (Spearman’s ρ = 0.44; *p* = 0.0203), indicating that states with higher online search interest in burns tended to have higher burn incidence rates (Fig. 3). Notable outliers (2023) included Santa Catarina, which had the highest search interest (score = 100) despite a moderate incidence rate (15.48 per 100,000), and Mato Grosso do Sul, which had below-average search interest (score = 69) with an above-average incidence (20.67 per 100,000). Conversely, states such as Goiás (score = 98, incidence = 53.77), Paraná (score = 82, incidence = 25.69), and Roraima (score = 67, incidence = 0.47) showed concordant patterns between search interest and hospitalization rates. In 2024, the correlation was non-significant (*p* = 0.31) (Fig. 3).

**Figure 3:**
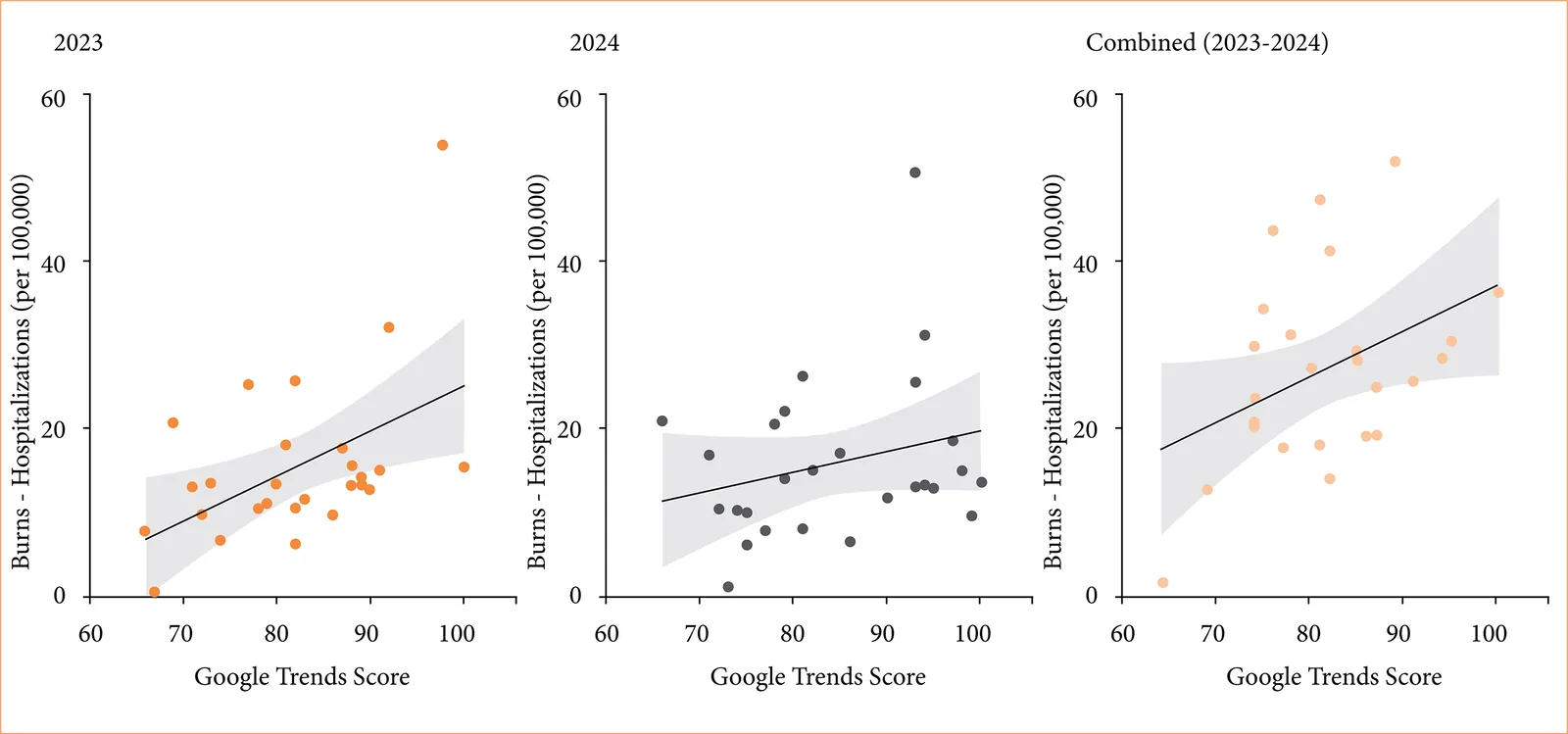
Scatterplots showing the correlation between Google Trends normalized scores and burn hospitalization incidence rates (per 100,000 inhabitants) across Brazilian states in 2023, 2024, and the combined 2023–2024 period. Each point represents one state. The trend line is shown for visual reference; statistical analysis used Spearman’s rank correlation (shaded area: 95% confidence interval). In the combinedpanel, Goiás (104.5 per 100,000) and Pernambuco (63.4 per 100,000) are outliers abovethe y-axis limit and are not displayed; both were included in the correlation analysis.

Source: Elaborated by the authors using data from DATASUS/SIH^15^, Google Trends^16^ and IBGE^17^.

### Spatial analysis

Spatial analysis showed no significant autocorrelation for hospitalizations in 2023 (Moran’s I = 0.07; pseudo *p*-value = 0.17) and 2024 (Moran’s I = 0.15; pseudo *p*-value = 0.08). Significant autocorrelation for GT scores was observed in 2023 (Moran’s I = 0.36; pseudo *p*-value = 0.004) and 2024 (Moran’s I = 0.25; pseudo *p*-value = 0.02). The bivariate autocorrelation of the score and hospitalization incidence showed no spatial correlation for 2023 and 2024 (Suppl. Mat.^23^).

### Exploratory analysis

Temporal analysis of burn hospitalizations (Fig. 4) identified a statistically significant cluster between June and October 2024, with 14,270 observed cases, and 13,474.88 expected (O/E ratio = 1.06). The model revealed a relative risk (RR) of 1.08 (Suppl. Mat.^23^).

**Figure 4:**
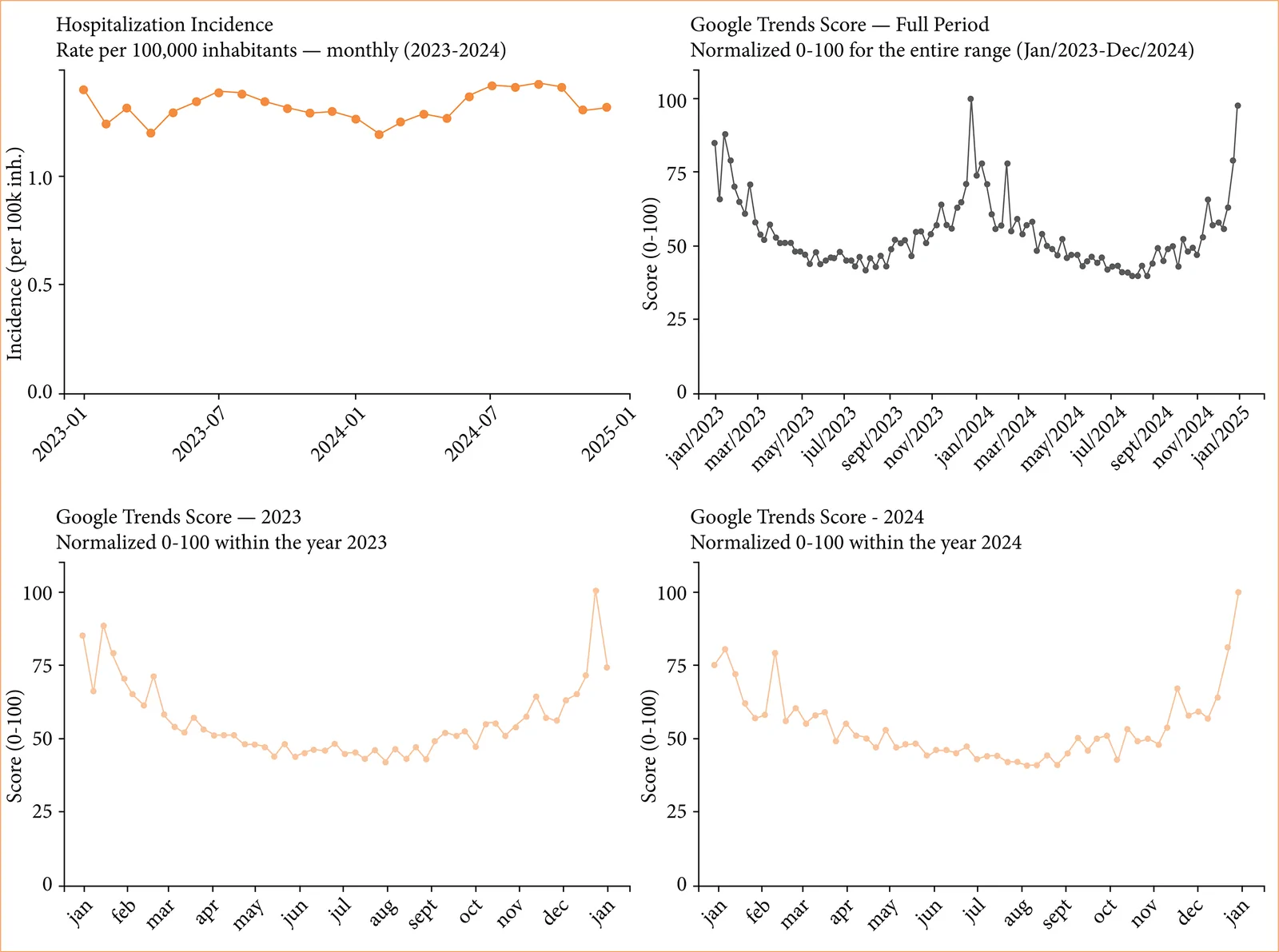
Time-series plots of burn hospitalization incidence (per 100,000 inhabitants) and Google Trends normalized scores (0–100) from January 2023 to December 2024. Upper panels show monthly hospitalization incidence (left) and overall search interest (right); lower panels show normalized Google Trends scores for 2023 (left) and 2024 (right) separately. Data were normalized within each period according to Google Trends methodology.

Source: Elaborated by the authors using data from DATASUS/SIH^15^, Google Trends^16^ and IBGE^17^.

### Sensitivity analysis

A sensitivity analysis was conducted using pooled 2023–2024 data. When the years were combined, the correlation between GT and burn incidence remained statistically significant and similar in magnitude (ρ = 0.44; *p* = 0.026). Spatial autocorrelation patterns were also consistent with year-specific analyses: GT showed significant clustering (I = 0.38, pseudo-*p* = 0.002), while burn hospitalizations incidence with GT score bivariate relationship showed no significant spatial structure (pseudo-p > 0.05) (Suppl. Mat.^23^). Bivariate spatial autocorrelation showed no significance (pseudo-*p* > 0.05).

### Related searches: Google Trends

Related searches showed that during the entire period, the highest relative search volume was observed for “*pomada queimadura*” (burn ointment, score = 100), followed by “*grau de queimadura*” (degree of burn, 77) and “*pomada para queimadura*” (ointment for burn, 75). Other frequently reported queries included “*queimadura de sol*” (sunburn, 64), “*bom para queimadura*” (good for burn, 50), “*bolha queimadura*” (burn blister, 42), “*o que é queimadura*” (what is a burn, 41), and “queimadura o que fazer” (burn what to do, 40).

Regarding rising queries, the following terms and respective growth rates in Brazil during the study period was obtained: “*qual é a melhor pomada para usar na queimadura*” (what is the best ointment to use for burn, +1,150%); “*como melhorar queimadura de sol rápido*” (how to improve sunburn quickly) (+400%); “*lagarta cachorrinho queimadura*” (caterpillar burn, +190%); “*qual é a melhor pomada para queimadura*” (what is the best ointment for burn, +80%); “*queimadura de água-viva fotos*” (jellyfish burn photos, +80%); “*queimadura o que fazer para aliviar a dor caseiro*” (burn what to do to relieve pain at home, +60%).

## Discussion

This study investigated whether online search interest in burns reflects the epidemiological distribution of burn-related hospitalizations across Brazilian states. Our findings suggest that the GT scores for “queimadura” remained consistently high in both years analyzed and demonstrated a moderate positive association with burn hospitalization incidence in 2023. However, this association was not statistically significant in 2024, indicating instability in the relationship between search interest and hospitalization incidence across years. Burn incidence varied substantially among states, with some states exhibiting high incidence without corresponding high search interest, and vice versa. Search interest exhibited significant spatial clustering, whereas hospitalization incidence did not show a similar spatial structure.

Temporal analysis revealed a significant cluster of hospitalizations from June to October 2024, suggesting an increased risk during this period. The data suggest that online search behavior may partially reflect the geographic and temporal burden of burns in Brazil, although this relationship appears to be dynamic rather than consistent over time. These findings should be regarded as hypothesis-generating rather than as validation of GT as a burn surveillance tool.

Digital search behavior following traumatic events is well documented, with patients and caregivers actively seeking online information regarding symptoms, treatment, and long-term recovery^24,25^. Infodemiological analyses of conditions like “whiplash syndrome” revealed structured search patterns driven by public interest in diagnosis and management^26^. In burn care, substantial gaps in first-aid knowledge among both the public and healthcare professionals drive reliance on external resources^27,28^. Consequently, the internet is the most frequently reported source of burn information, often surpassing formal education^28^, as patients routinely supplement professional guidance with independent online searches^29^. While traditional media and cultural knowledge remain relevant^30^, burn survivors increasingly utilize social platforms for information exchange and peer support^31^. Collectively, these findings suggested that traumatic events elicit structured, multi-source information-seeking behaviors, providing a behavioral foundation for the infodemiology research in burns.

Related search data offer insight into the informational priorities of the population seeking burn-related content. The predominance of management-oriented terms over prevention-focused queries suggests a reactive information-seeking pattern, in which individuals search after injury rather than in anticipation of risk. This behavioral profile may inform the design of health education campaigns by identifying content gaps and preferred entry points for preventive messaging. Notably, two rising queries referenced envenomation-related skin injuries caused by jellyfish and caterpillars, representing a distinct injury mechanism that may warrant dedicated investigation in future infodemiology studies.

A direct comparison between Figs. 1 and 2 illustrates the spatial decoupling central to this study’s findings. Burn hospitalization incidence showed no significant spatial autocorrelation in either year, whereas GT scores clustered significantly across both (Fig. 4). This divergence suggests that online search behavior is not solely driven by burn incidence but is also shaped by structural factors—including disparities in internet access, socioeconomic development, and Brazilian Unified Health System coverage—that are distributed differently across states than burn injury burden itself.

Temporal analysis identified a statistically significant hospitalization cluster between June and October 2024, coinciding with *festa junina* celebrations, during which exposure to bonfires and fireworks increased substantially—a well-documented seasonal pattern in the national literature^32^. Notably, this rise in hospitalizations was not accompanied by a proportional increase in search interest. Rather than inferring campaign failure, this decoupling highlights a gap in population-level health information behavior that warrants dedicated cross-sectional investigation into how different groups access and process burn-related health information in Brazil.

A distinct pattern emerges at year-end: GT activity spiked markedly from late 2024 without a corresponding rise in hospitalizations. Unlike the *festa junina* period, fireworks-related burn hospitalizations in Brazil do not peak around New Year’s Eve. The year-end search spike therefore likely reflects preventive or reactive information-seeking rather than increased burn incidence, though its specific drivers cannot be determined from the available data and merit further investigation.

The limitations of this study must be acknowledged when interpreting these findings. First, the ecological design precludes causal inference, and associations observed at the state level may not reflect individual-level relationships (*i.e.*, ecological fallacy). Second, disparities in internet access across Brazilian states may influence the investigation of the relationship between online search interest and burn incidence. Finally, the study was conducted in a single country, which limits the generalizability of the findings to other settings. Future work should investigate patient-level digital behavior following burn injuries, evaluating key platforms, search terms, and content types, to inform the design of subsequent population-level studies.

## Conclusion

This study offered an exploratory examination of whether GT search interest may partially reflect the distribution of burn hospitalizations across Brazilian states. The findings—a moderate correlation in 2023, significant spatial clustering of search scores, and a temporal hospitalization cluster during *festa junina*—are suggestive rather than conclusive, and their inconsistency across years highlights the preliminary nature of this evidence. Whether online search behavior can meaningfully complement administrative burn surveillance data remains an open question.

This study does not establish GT as a validated surveillance tool; it proposes it as a signal worth investigating further. Future studies incorporating patient-level behavioral data, and longer time series are better positioned to determine how robust and clinically useful this approach may prove in Brazil and other regions.

## Data Availability

The data are available in a data repository: https://doi.org/10.6084/m9.figshare.34029465.
